# Exercise-induced arterial hypertension - an independent factor for hypertrophy and a ticking clock for cardiac fatigue or atrial fibrillation in athletes?

**DOI:** 10.12688/f1000research.4001.1

**Published:** 2014-05-12

**Authors:** Roman Leischik, Norman Spelsberg, Hiltrud Niggemann, Birgit Dworrak, Klaus Tiroch

**Affiliations:** 1 Department of Cardiology, Section of Prevention, Health Promotion and Sports Medicine, Faculty of Health, School of Medicine, Witten/Herdecke University, Hagen, 58095, Germany; 2 Department of Cardiology, Faculty of Health, School of Medicine, Witten/Herdecke University, Helios Hospital Wuppertal, 42117, Germany

## Abstract

**Background** **:** Exercise-induced arterial hypertension (EIAH) leads to myocardial hypertrophy and is associated with a poor prognosis. EIAH might be related to the “cardiac fatigue” caused by endurance training. The goal of this study was to examine whether there is any relationship between EIAH and left ventricular hypertrophy in Ironman-triathletes.

**Methods:** We used echocardiography and spiroergometry to determine the left ventricular mass (LVM), the aerobic/anaerobic thresholds and the steady-state blood pressure of 51 healthy male triathletes. The main inclusion criterion was the participation in at least one middle or long distance triathlon.

**Results**: When comparing triathletes with LVM <220g  and athletes with LVM >220g there was a significant difference between blood pressure values (BP) at the anaerobic threshold (185.2± 21.5 mmHg *vs.* 198.8 ±22.3 mmHg, p=0.037). The spiroergometric results were: maximum oxygen uptake (relative VO _2_max) 57.3 ±7.5ml/min/kg *vs*. 59.8±9.5ml/min/kg (p=ns). Cut-point analysis for the relationship of BP >170 mmHg at the aerobic threshold and the probability of LVM >220g showed a sensitivity of 95.8%, a specificity of 33.3%, with a positive predictive value of 56.8 %, a good negative predictive value of 90%. The probability of LVM >220g increased with higher BP during exercise (OR: 1.027, 95% CI 1.002-1.052, p= 0.034) or with higher training volume (OR: 1.23, 95% CI 1.04 -1.47, p = 0.019). Echocardiography showed predominantly concentric remodelling, followed by concentric hypertrophy.

**Conclusion**: Significant left ventricular hypertrophy with LVM >220g is associated with higher arterial blood pressure at the aerobic or anaerobic threshold. The endurance athletes with EIAH may require a therapeutic intervention to at least prevent extensive stiffening of the heart muscle and exercise-induced cardiac fatigue.

## Introduction

**Figure 1.**
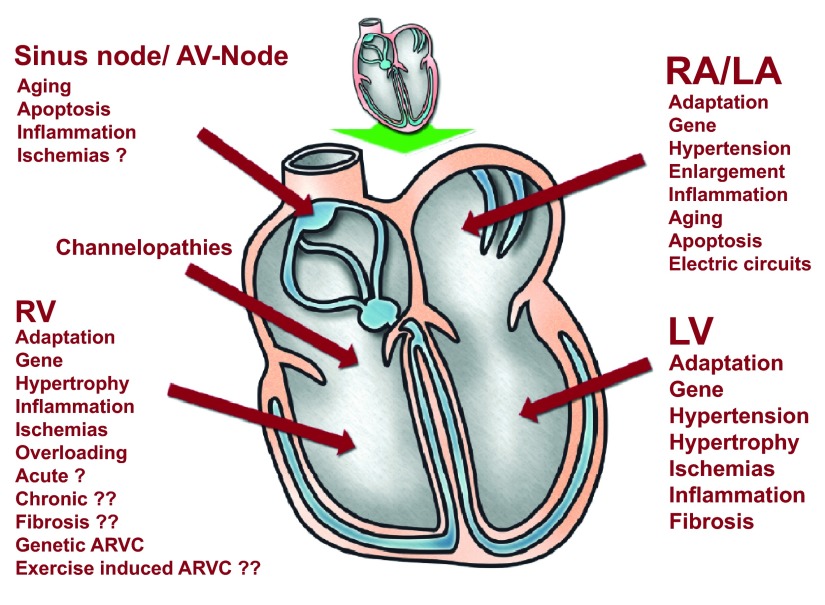
Factors which affect cardiac structures and function during exercise. This figure shows the factors with possible negative influence on myocardium like inflammation, fibrosis etc. It demonstrates the possible complexity of different actions.

**Figure 2.**
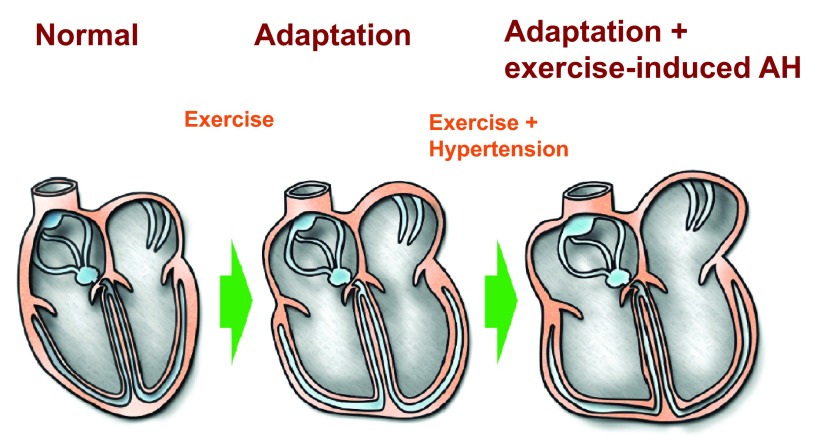
Scheme of possible adaptation of cardiac cavities in endurance sport and possible pathological enlargement/hypertrophy in case of exercise-induced arterial hypertension. Right and left atrium have more connective tissue construct as muscular ventricular chambers and more affinity for pathological enlargement in case of pressure overload.

Myocardial hypertrophy in hypertensive patients has a negative influence on long-term prognosis ^1^, cardiac arrhythmias and mortality ^2, 3^. Myocardial hypertrophy in otherwise healthy, non-hypertensive individuals can be caused by exercise-induced arterial hypertension (EIAH) ^4– 6^ and may also result in poor prognosis ^7^. Moreover, exercise-induced hypertrophy may cause sudden cardiac death in athletes ^8^. However, myocardial hypertrophy induced by extensive exercise presents so called “normal diastolic” function, which might be a result of “physiological” adaptation ^9, 10^. EIAH or elevated blood pressure values during exercise might have a “negative” impact on cardiac function in athletes and might be one of the important factors causing “exercise-induced” cardiac fatigue ( Figure 1). Our hypothesis is provocative, but this suggestion might become important for many professional and leisure athletes ( Figure 2).

Physical activity in the general population is of fundamental importance ^11, 12^. The role of EIAH in normotensive adult athletes ^13^ or healthy men is currently under discussion ^6, 14^. It is unclear how far endurance sport can influence a “negative remodelling” of the athlete’s heart ^15^. The dosage of exercise bouts which causes cardiac injury ^18, 19^, and the “true pathologic values” of EIAH are unknown or under debate ^6^.

Endurance sport is linked to cardiac injury ^20^. In individual cases, long term training might lead to arrhythmias ^21^, atrial fibrillation ^22, 23^ or myocardial fibrosis ^16, 24^ and early sudden cardiac death ^24– 26^, female athletes are less commonly affected ^26, 27^.

The type of sport discipline has also an influence on the type of hypertrophy. Some authors distinguish the strength-trained heart and an endurance-trained heart ^10, 28^. Further factors that might influence exercise-induced hypertrophy are genetic factors ^29, 30^, gender ^31^, environmental factors ^32^, endocrine factors ^33^ and arterial hypertension ^34^.

In this study, we examined the impact of EIAH on cardiac hypertrophy in 51 normotensive (at rest) healthy Ironman athletes with long daily training times.

## Materials and methods

The influence of EIAH on cardiac hypertrophy was examined in 51 male triathletes (mean age 37.2, Table 1) who finished an Ironman 70.3 (n=17, 1.9km swim, 90km bicycle ride and 21,1km run) or Ironman full distance (n=34/3.8km swim, 180km bicycle ride and/42.2km run). The training habits were similar for both the 70.3 and long distance Ironman. The minimum training-time was two years. All triathletes have been examined by spiroergometry and echocardiography. There is no consensus about the value of systolic BP that determines EIAH ^6^. According to the literature, EIAH is described as systolic BP >210mmHg for males and >190mmHg for females, as maximal values during exercise ^5^. In our study, the absolute values primarily were not defined. Odds ratios analysis was used to calculate the probability of elevated blood pressure and hypertrophy. The estimation of sensitivity/specificity to detect the boundaries of the EIAH and the positive or negative predictive values for the blood pressure boundaries should be performed during the study. Our interest was directed to the aerobic and anaerobic threshold, because this is a constant level of blood pressure maintained during training or competitions.

**Table 1.** Anthropometric and echocardiographic data. The blue coloured area shows the anthropometric data of the two groups with different LVM. The orange coloured data are echocardiographic data of the left ventricle. The green coloured data are the Doppler-flow data. The last two lines are the data of the right ventricle.

	LVM <220g			LVM >220g			p-value
	n	mean	sd	n	mean	sd	Mann-Whitney-U-Test
Age (years)	27	37.2	10.7	24	38.3	13.5	0.947
Weight (kg)	27	182.1	6.30	24	182.5	7.1	0.932
Size (cm)	27	75.0	6.20	24	78.2	11.3	0.231
BMI (kg/m ^2^)	27	22.6	1.60	24	23.4	2.0	0.206
BSA (m ^2^)	27	1.95	0.11	24	1.99	0.18	0.395
%body fat	27	12.3	3.4	24	12.4	3.9	0.828
Aorta (cm)	27	2.9	0.4	24	3.0	0.3	0.236
Left atrium (cm)	27	2.46	0.27	24	2.64	0.27	0.020
LAESV* (ml)	27	27.7	8.00	24	30.7	7.5	0.098
IVS diastolic (cm)	27	1.16	0.10	24	1.31	0.12	0.000
IVS systolic (cm)	27	1.57	0.13	24	1.78	0.16	0.000
PWD diastolic (cm)	27	1.13	0.08	24	1.32	0.13	0.000
PWD systolic (cm)	27	1.59	0.1	24	1.83	0.14	0.000
Relative wall thickness	27	0.48	0.06	24	0.53	0.07	0.055
LVEDD (cm)	27	4.7	0.4	24	5.0	0.3	0.003
LVESD (cm)	27	4.7	0.4	24	5.0	0.3	0.003
LVM (g)	27	185.3	19.3	24	254.1	27.0	0.000
LVM (g/m ^2^)	27	95.3	10.1	24	128.8	17.6	0.000
LVEDV (ml)	27	132.7	18.8	24	145.0	24.4	0.086
LVESV (ml)	27	50.6	7.9	24	55.0	11.4	0.234
SV (ml)	27	82.0	11.9	24	89.9	15.3	0.059
EF (%)	27	62.5	2.0	24	61.6	2.9	0.212
LVOT V _max_ (m/s)	27	0.80	0.13	24	0.80	0.13	0.917
MV E _max_ (m/s)	27	0.54	0.1	24	0.51	0.09	0.196
MV A _max_ (m/s)	27	0.37	0.07	24	0.36	0.05	0.857
MV E/A Ratio	27	1.51	0.35	24	1.43	0.27	0.313
RV parasternal	27	3.1	0.1	24	3.30	0.1	0.000
RV AFC%	27	32.8	1.8	24	34.2	2.4	0.047

Mean = mean value. sd = standard deviation. BMI = body mass index. BSA = body surface area. LAESV = left atrial endsystolic volume. IVS = interventricular septum. PWD = diastolic left ventricular posterior wall thickness. RWT: relative wall thickness: (2xPWD/LVEDD). LVEDD = left ventricular end-diastolic diameter. LVM = left ventricular mass. LVEDV: left ventricular enddiastolic volume. LVESV: left ventricular endsystolic volume. SV: Stroke volume. EF: Ejection fraction in %. LVOT = left ventricular outflow tract. MV: Mitral valve. parasternal: right ventricular diameter in 2D parasternal view. RV AFC%: right ventricular area fractional change.

A Vivid 7 model echocardiograph manufactured by general Electric was used for the examinations. The Ergobike 8I manufactured by Daum and the Metalizer 3B produced by Cortex were used for the spiroergometric examination ^35^.

The assessment of each triathlete was performed in 2011 and 2012 on the same day with the echocardiography first followed by spiroergometry. The spiroergometry was performed as follows: the stress test (exercise bike) was conducted in stages after successful gas and volume calibration: 50W for 3 minutes, 100W for further 3 minutes and thereafter increased by another 30W for 3 minutes (ramp-test). The test ended when the subject could no longer maintain the predefined rpm of 90 or if the subject was exhausted.

The echocardiographic analysis was conducted according to general recommendations ^36, 37^. The formula recommended by the American Society of Echocardiography (ASE) was used for calculate the muscle mass. Enddiastolic LV-volume (EDV) and Endsystolic LV-volume (ESV) were determined monoplane after the modified Simpson method ^36^.

The spiroergometric analyses were conducted according to previously published protocols ^38, 39^: VAT (ventilatory aerobic threshold) was determined as the first non-linear increase of the ventilatory equivalent for oxygen without simultaneous increase of the ventilatory equivalent for CO _2_, and RCP (respiratory compensation point: anaerobic threshold) was determined as simultaneous non-linear increase of both ventilatory equivalents according to previous recommendations ^38, 39^.

VO _2_max was registered as the highest average value of oxygen absorption over 30 seconds.

### Statistical analysis

The entire statistical analysis plan was designed as follows: Stata/IC 13.1 for Windows was used for data preparation and statistical analysis. The Mann-Whitney-U-Test was used to compare the groups with LVM >220g and LVM <220g. Odds Ratios were calculated to measure the association between blood pressure, training habits and the probability of LVM >220g. Since these exposure variables are quantitative variables, an approximate estimate of the log odds-ratio for a one-unit increase in exposure and a 1-degree-of-freedom test for trend were calculated. All statistical tests were two-sided with a signficance level of 0.05.

In addition, sensitivity, specificity, positive and negative predictive values as well as the proportion of correctly classified participants were calculated for each possible cut-point of blood pressure to describe the performance of blood pressure as a “diagnostic test” for LVM >220g.

## Results

### Anthropometry and echocardiography

**Figure 3.**
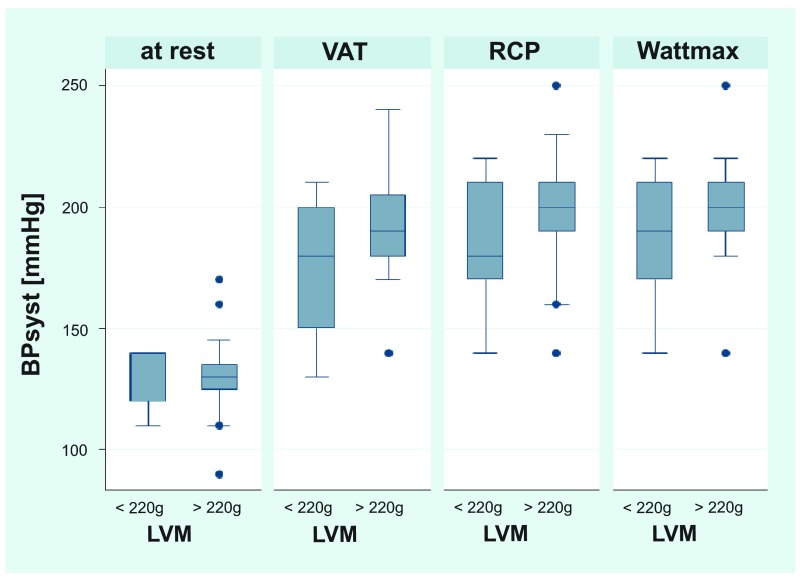
Blood pressure values at rest and at different exercise levels in two groups of triathletes with different LVM. The group with LVM >220 shows significant higher systolic blood pressure (BP) values at the aerobic threshold (VAT), anaerobic threshold (RCP) and at the maximum exercise-level (Wattmax). BP = Blood pressure. LVM = left ventricular mass. VAT = ventilatore aerobic threshold. RCP = respiratory compensation point. Wattmax = Maximum exercise-level.

Anthropometric baseline data of triathletes are listed in Table 1. The blood pressure values of the two groups (LVM <220g and LVM >220g) are visualized in Figure 3 (for exact values see Table 2). In Figure 3 one can see that triathletes with LVM >220g have higher blood pressure values at ventilatory aerobic threshold (VAT) and anaerobic threshold (RCP) and at maximum achieved Watt-level (Wattmax). The results showed myocardial hypertrophy in most participants and were classified as according to Lang *et al.* ^36^. Normal morphology was found in three triathletes, eccentric hypertrophy was shown in one athlete, concentric remodelling was observed in 26 triathletes and concentric hypertrophy in 21. Right ventricular remodelling or other pathological findings of the right ventricle were not found in any of the athletes. Left ventricular function was good in all triathletes (EF >55%). All relevant echocardiographic values are shown in Table 1. All further parameters are shown in Table 2, sorted according to the p-value.

**Table 2.** Performance, BP and training parameters depending on LVM, sorted according to p-value. Triathletes in the group with LVM >220g have significant longer training times and distances on bike, longer overall training times ( **Mann-Whitney-U-Test**).

Further parameters	LVM <220g			LVM >220g			p-value
	n	Mw	SD	n	Mw	SD	Mann-Whitney-U-Test
abs. VO _2 AerobicThreshold_	27	3.2	0.5	24	3.7	0.5	0.001
abs.VO _2 AnaerobicThreshold_	27	3.6	0.5	23	4.2	0.7	0.001
Tr-distance bike/week	27	190.3	65.8	24	250.2	60	0.004
Watt _AnaerobicThreshold_	27	295.6	43.5	23	332.2	51.3	0.014
rel. VO _2 AerobicT. ml/kg/min_	27	42.5	7.8	24	48.2	7.9	0.017
Watt _AerobicThreshold_	27	265.6	46.6	24	301.3	53.4	0.023
%VO _2max AnaerobicThreshold_	27	85	10.5	23	90.6	9	0.026
Tr-time bike	27	7	2.2	24	8.6	2.4	0.034
Tr-time overall	27	15.7	2.7	24	17.8	3.3	0.035
BPs _AnaerobicThreshold_	27	185.2	21.5	24	198.8	22.3	0.037
Watt _max_	27	336.7	41.9	24	363.8	56.6	0.042
%VO _2max AerobicThreshold_	27	74.3	12.1	24	81	8.9	0.046
Tr-time swim/week	27	3.2	1.2	24	3.8	1.4	0.049
rel.VO _2 AnaerobicThreshold_	27	48.4	7.2	23	54.4	9.9	0.054
BPs _Wattmax_	27	188.1	20.4	24	199.6	19.9	0.055
BPs _AerobicThreshold_	27	178	24.6	24	192.9	20.5	0.056
abs. VO _2max_	27	4.3	0.5	24	4.6	0.8	0.059
Tr-distance swim/week	26	6.9	3.5	24	8.7	4.2	0.090
BPs _Rest_	27	125.4	10.8	24	130.8	15.7	0.105
Triathlon since years	27	7.4	4.8	24	11	7.7	0.142
rel. VO _2max ml/min/kg_	27	57.3	7.5	24	59.8	9.5	0.281
HR rest	27	60.3	5.5	24	58.9	8	0.328
Tr-distance run/week	27	51.4	14.6	24	53.8	12.1	0.355
HR _max_	27	179.4	10.6	24	176.2	11.5	0.385
Watt _max/kg_	27	4.5	0.6	24	4.7	0.7	0.433
HR _AerobicThreshold_	27	150	14.8	24	152.7	12.6	0.503
IVRT	27	101.3	23.3	24	103.1	16.5	0.515
HR _AnaerobicThreshold_	27	162.7	12.5	23	163.7	12	0.599
BPd _Wattmax_	27	79.8	9.2	24	79.8	10.7	0.891
BPdiastol _RCP_	27	78	7.9	24	78.3	11.3	0.913
Tr-time run/week	27	4.9	1.5	24	4.9	1.2	1.000
BPd _AerobicThershold_	27	78.5	9.1	24	78.8	10.8	1.000

Tr = training. BP = Blood Pressure BPs _AnaerobicThreshold_ = systolic blood pressure at the anaerobic threshold BPs _Wattmax_ = systolic blood pressure at the maximum power output time rel. VO _2RCP_ = relative oxygen uptake at the anaerobic threshold rel. VO _2max ml/min/kg_ = relative maximal oxygen uptake IVRT = Isovolumetric relaxation time Watt _max_ = maximum power output

### Spiroergometry/physiological performance/blood pressure values

Oxygen uptake, ergometer performance and heart rate with VAT, RCP and at peak capacity are shown in Table 3. Participants with LVM >220g achieved at all thresholds and at maximum level higher power output values. Relative oxygen uptake values were slightly higher in the group with LVM >220g but not significant different at the maximum stage of loading. Spiroergometric maximum oxygen uptake (relVO _2_max) was 57.3±7.5 ml/min/kg vs. 59.8±9.5 ml/min/kg (p=n.s.) for LVM <220g *vs*. >220g, respectively.

**Table 3.** Heart rate, oxygen uptake and performance in both groups of triathletes with different LVM. The table is divided in three main blocks: the first block reflects data at the aerobic threshold, the second one at the anaerobic threshold and the last one at the maximum exercise stage.

	LVM <220g			LVM >220g			p-value
	n	Mv	sd	n	Mv	sd	Mann-Whitney-U-Test
	VAT (ventilatory aerobic threshold)						
HR	27	150.0	14.8	24	152.7	12.6	0.503
aVO _2_	27	3.2	0.5	24	3.7	0.5	0.001
rVO _2_	27	3.2	0.5	24	3.7	0.5	0.001
%VO _2max_	27	74.3	12.1	24	81.0	8.9	0.046
Watt	27	265.6	46.6	24	301.3	53.4	0.023
	RCP (respiratory compensation point = anaerobic threshold)						
HR	27	162.7	12.5	24	163.7	12.0	0.599
aVO _2_	27	3.6	0.5	24	4.2	0.7	0.001
rVO _2_	27	48.4	7.2	24	54.4	9.9	0.054
%VO _2max_	27	85.0	10.5	24	90.6	9.0	0.026
Watt	27	295.6	43.5	24	332.2	51.3	0.014
	Peak capacity						
HR	27	179.4	10.6	24	176.2	11.5	0.385
aVO _2_	27	4.3	0.5	24	4.6	0.8	0.059
rVO _2_	27	57.3	7.5	24	59.8	9.5	0.281
Watt	27	336.7	41.9	24	363.8	56.6	0.042

Mv = Mean value; sd = standard deviation, aVO2 = absolute oxygen uptake in L/min, rVO2 = relative oxygen uptake in ml/min/kg, % point of the overall exercise-test HR = heart rate, Watt = power output

Table 4 shows the cut-point analysis for blood pressure values and the probability of development of LVM >220g. BP values over 180mmHg at the aerobic threshold might define the athletes at risk of developing LVM >220g and a possible further cardiac fatigue.

**Table 4.** Cut-point analysis for the relationship of blood pressure at the aerobic threshold and the probability of LVM >220g.

BP	Sens.%	Spec. %	PPV %	NPV %	Correct %
130	100	0	47.1	-	47.1
140	100	3.7	48	100	49
145	95.8	7.4	47.9	66.7	49
150	95.8	11.1	48,9	75	51
160	95.8	25.9	53.5	87.5	58.8
170	95.8	33.3	56.1	90	62.7
180	87.5	40.7	56.8	78.6	62.7
190	62.5	55.6	55.6	62.5	58.8
200	45.8	66.7	55	58.1	56.9
210	25	85.2	60	56.1	56.9
220	12.5	100	100	56.3	58.8
240	4.2	100	100	54	54.9

### Left ventricular hypertrophy

According to the values reported by Devereux *et al.* ^40^ and Bove *et al.* ^41^, we divided the triathletes in two groups: group 1 (LVM >220g) and group 2 (LVM <220g) to assess the possible reasons for left ventricular hypertrophy. The significant differences between the two groups are shown in Table 1 and Table 2. In summary, left ventricular mass (<220g *vs.* >220g) is associated with significantly different blood pressure values at the anaerobic threshold (185.2±21.5mmHg *vs.* 198.8±22.3mmHg, p=0.037).

**Figure 4.**
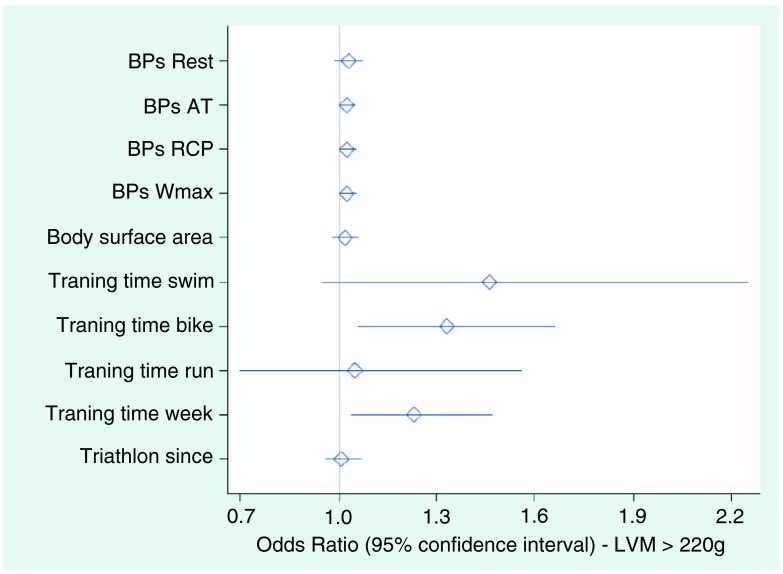
Odds ratios analysis for probability of LVM. This figure is based on the values reported in Table 5. Values above 1.0 show a significant relationship between bike training times and overall training times and LVM >220g.

The probability of dependent factors for LV-hypertrophy was calculated by odds ratios ( Table 5). Odds ratios analysis showed a significant relationship between the arterial pressure values during exercise (significant p-values at the aerobic and anaerobic threshold in Table 5). The significant values are bold in Table 5. A further relationship was found between bike training times and overall training times and LVM >220g ( Figure 4). Values above 1.0 show this significant relationship.

**Table 5.** Odds Ratios with 95% confidence intervals (CI) for probability of LVM >220g. The significant p-values at the aerobic and anaerobic threshold are in bold.

	LVM <220g (n=27)		LVM >220g (n=24)					
	mean	SD	mean	SD	OR	95%-CI		p-value
BPs _Rest_	125.4	10.8	130.8	15.7	1.031	0.989	1.074	0.148
BPs _AerobicT_	178.0	24.6	192.9	20.5	**1.027**	**1.003**	**1.051**	**0.025**
BPs _AnaerobicT_	185.2	21.5	198.8	22.3	**1.027**	**1.002**	**1.052**	**0.034**
BPs _Wattmax_	188.1	20.4	199.6	19.9	1.027	1.000	1.054	0.050
BSA	194.7	10.6	198.7	17.9	1.02	0.98	1.06	0.328
Tr-time swim	3.2	1.2	3.8	1.4	1.46	0.95	2.25	0.081
Tr-time bike	7.0	2.2	8.6	2.4	**1.33**	**1.06**	**1.66**	**0.015**
Tr-time run	4.9	1.5	4.9	1.2	1.05	0.70	1.56	0.823
Tr-time overall	15.7	2.7	17.8	3.3	**1.23**	**1.04**	**1.47**	**0.019**
Triathlon since years	14.5	9.0	15.7	10.3	1.01	0.96	1.07	0.654

mean = mean value. BSA = Body Surface Area Tr = Training BPs = systolic blood pressure AerobicT = Aerobic threshold AnaerobicT = Anaerobic Threshold

Data of exercise-induced arterial hypertension in triathletes Anthropometry parameters, training habits, echocardiography and spirometry data of 51 healthy male triathletes who completed an Ironman 70.3 or an Ironman full distance race are shown. D = Ironman Distance, LD Long distance / MD = Ironman 70.3, A= Age, G= gender, We = Weight, H = Height, BSA = body surface area, %BF = %body fat, Tts = Training time swim, Tds= Training distance swim, Ttb= Training time bike, Tdb= Training distance bike, Ttr= Training time run, Tdr= Training distance run, Ttt= Total training time, Ts= Triathlon since, sbT= Sport before Triathlon, sbTs= Sport before triathlon since, HRmax= Heart Rate at exertion, HRVAT= Heart rate at ventilator anaerobic threshold, HRRCP= Heart rate at respiratory compensation point, HRLAM= Heart rate at Lactate threshold 4,0mmol/l (Mader), HRLAD= Heart rate at Lactate threshold according to Dickhuth, HRLAI= Heart rate at first nonlinear increase of blood lactate, Abs VO2max= Maximum oxygen uptake L/min, Abs. VO2VAT= Oxygen uptake at ventilatory anaerobic threshold L/min, Abs. VO2RCP= Oxygen uptake at respiratory compensation point L/min, Rel VO2max= Maximum oxygen uptake relative to body weight mlL/min/kg, Rel VO2VAT= Oxygen uptake at ventilator anaerobic threshold relative to body weight mlL/min/kg, Rel VO2RCP= Oxygen uptake at respiratory compensation point relative to body weight mlL/min/kg, %VO2maxAT= Oxygen uptake at ventilator anaerobic threshold as percentage of maximum oxygen uptake, %VO2maxRCP= Oxygen uptake at respiratory compensation point as percentage of maximum oxygen uptake, VEmax= Maximum minute ventilation, O2HFmax= Maximum O2 Pulse, RERmax= Maximum Respiratory Exchange Ratio, BLCmax= Blood lactate concentration at exertion, Wmax= Maximum ergometer performance (Watt), WAT= Ergometer performance at ventilator anaerobic threshold, WRCP= Ergometer performance at respiratory compensation point, WLAM= Ergometer performance at Lactate threshold 4,0mmol/l (Mader), WLAD= Ergometer performance at Lactate threshold according to Dickhuth, WLAI= Ergometer performance at first nonlinear increase of blood lactate, Wmax/kg= Maximum ergometer performance in relation to body weight (Watt/kg), BPsRest= Systolic blood pressure at rest, BPdRest= Diastolic blood pressure at rest, BPsVAT= Systolic blood pressure at ventilator anaerobic threshold, BPdVAT= Diastolic blood pressure at ventilator anaerobic threshold, BPsRCP= Systolic blood pressure at respiratory compensation point, BPdRCP= Diastolic blood pressure at respiratory compensation point, BPsWmax= Systolic blood pressure at exertion, BPdWmax= Diastolic blood pressure at exertion, Ao= Aortic root dimension, LA= Left atrial diameter, IVSd= Inter-ventricular septum in diastole, LVIDd= Left ventricular internal diameter in diastole, LVPWd= Left ventricular posterior wall in diastole, %IVS= Percentage of thickening of the inter-ventricular septum form diastole to systole, LVIDs= Left ventricular internal diameter in systole, %FS= Fractional shortening, LVPWs= Left ventricular posterior wall in systole, LV Mass (ASE) = left ventricular mass according to the ASE recommended formula, LVEDV MOD A4c ml= Left ventricular end-diastolic volume calculated according to the Simpson method in apical 4 chamber view, LVESV MOD A4c ml= Left ventricular end-systolic volume calculated according to the Simpson method in apical 4 chamber view, LVEF MOD A4c= Left ventricular ejection fraction calculated according to the Simpson method in apical 4 chamber view, SV Mod A4C ml= Stroke volume calculated according to the Simpson method in apical 4 chamber view, EF Biplan= Left ventricular ejection fraction calculated according to the Simpson method in apical 4 and 2 chamber view, LVEDV MOD BP ml= Left ventricular end-diastolic volume calculated according to the Simpson method in apical 4 and 2 chamber view, LVESV MOD BP ml= Left ventricular end-systolic volume calculated according to the Simpson method in apical 4 and 2 chamber view, MV E Max m/s= Mitral valvular E-Wave m/s, MV A Max m/s= Mitral valvular A-Wave m/s, MV E/A Ratio= Mitral valvular E/A Ratio, LVOT Vmax= Left ventricular outflow tract maximum velocity in PW-Doppler, HR Rest= Heart frequency at rest, RWT= Relative wall thickness, RV parasternal= Right ventricular diameter in the parasternal long axis, RVDA = Right venrticular area in diastole, RVSA= Right ventricular area in systole, RV FAC= Area change fraction RVSAx100/RVDA.

## Discussion

**Figure 5.**
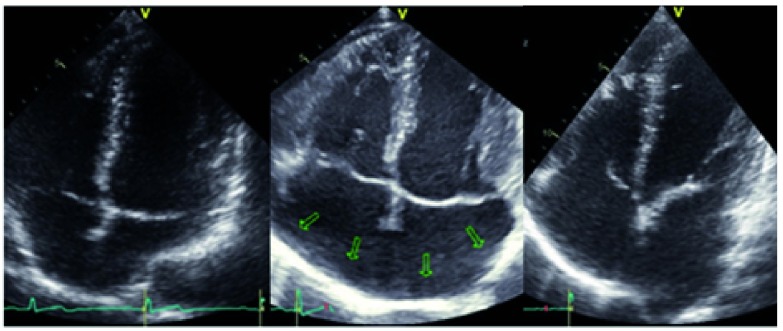
Pathological cardiac remodelling (especially of right and left atrium) of a 48 years old triathlete with EIAH. In 2011, the participant showed a normal size of the right and left atrium. An atrial enlaragement occured 2014 (arrows) after a period of high intensity training (echocardiography in 4 chamber view during atrial fibrillation). Even after cardioversion (2014) into sinus rhythm (7 days in sinus rhythm) he retains larger atrial cavities as in 2011.

The most interesting finding of this study is that myocardial hypertrophy depends on exercise-induced arterial hypertension. This confirms the results described by Douglas *et al.* ^13^ and Longas-Tejero *et al.* ^42^, who found a hypertensive response to exercise in eight of 37 healthy athletes (18 soccer players, 12 mountain climbers and seven canoeists). In this cited study, athletes with EIAH showed higher LVM (205g/m ^2^) compared to those without exaggerated blood pressure response to exercise (143g/m ^2^). There is no consensus about the value of systolic blood pressure that constitutes EIAH ^6^. According to our study, it seems that a systolic BP value >180mmHg at the aerobic threshold indicates exercise-induced arterial hypertension. So far, the boundaries for EIAH have never been estimated. In this study, we have chosen the aerobic threshold as the measuring point because the majority of the triathlete’s training is carried out at this level. When the hypertensive BP value is reached, we should analyse whether a careful low dosage treatment might be beneficial (for example with ACE inhibitors or AT _1_-blockers). The goal of such therapy would be to cut the blood pressure peaks (bouts) during training or competitions and avoid an increase of stiffness of the aorta ^43^ or LV-hypertrophy in people at risk. Raised BP bouts can lead to pathological enlargement of atrial dimensions in athletes ( Figure 2 and Figure 5). Enlargement of the left atrium may lead to atrial fibrillation and higher activity of electric circuits. There are no clear statements or guidelines regarding the role of EIAH in the daily practice of sports medicine ^44^. This manuscript may encourage a discussion about this important issue. The possible impact of EIAH on cardiac structures in triathletes is shown in Figure 2. A specific case of cardiac remodelling is shown in Figure 5. In this Figure are shown normal heart cavities of a triathlete with EIAH in 2011 and massive atrial enlargement in 2014. Exercise-induced hypertension was often discussed in the 1990s ^4, 5, 7^ reflecting the results of the Framingham Study ^5^. The negative role of EIAH in non-athletic men is relatively clear ^6^, but the impact on athletes needs to be discussed and the “pathological range” of EIAH should be evaluated. Exercise-induced hypertension promotes myocardial hypertrophy ^4^ and increases cardiovascular risks ^7^ in normotensive men. Athletes with EIAH are in similar way “persons at risk” and may develop a pathological cardiac chamber enlargement and atrial fibrillation, but have less “cardiovascular risk” because of the healthier life style and the positive impact of sport in the development of arteriosclerotic complications.

### Cardiac adaptation to exercise, left ventricular hypertrophy and sudden cardiac death

The specific endurance training of triathletes leads to physiological changes of performance parameters ^45^ and also results in changes in cardiac function or heart structures ^46^. This adaptation is linked to the nature and magnitude of the physical exercise ^31^. The physiological adaptation is a “harmonic increase in size” of a healthy heart caused by physical activity ^47^. The term “athlete’s heart” ^9, 31^ has been known since 1899 ^48^. Cardiovascular adaptations to exercise have been systematically defined according to the type of endurance training.

Concentric hypertrophy in triathletes has already been described ^49, 50^. Douglas *et al.* ^50^ suggested that athletes develop hypertrophy possibly due to the systolic blood pressure increase under exercise, which could be explained by the frequency of the training. Diastolic function was shown to be normal under those conditions.

In the present study, odds ratio analysis showed a significant relationship of myocardial thickening to exercise-induced blood pressure. It can be assumed that training over an extended period with exercise-induced blood pressure elevation can lead to hypertrophy in a similar way to that found in pathological conditions with arterial hypertension. Concentric remodelling was found in 26 cases and concentric hypertrophy in 21 cases. Concentric remodelling and concentric hypertrophy occurs more often in male athletes ^31^. Different authors have concluded that strength training predominantly leads to concentric hypertrophy and endurance training to eccentric hypertrophy ^28^. In this study concentric remodelling was observed most frequently. George *et al.* ^31^ reported that the expected pattern of eccentric enlargement was replaced by a pattern of concentric or symmetric enlargement in groups of highly trained athletes. Generally, the adaptation of the cardiac mass seems not to depend on the type of conditioning ^31^. In 1989, Douglas *et al.* ^13^ published a comparison of 36 triathletes with 17 normal controls and 15 arterial hypertension patients. They determined that triathletes undergo cardiac adaptations similar to pressure overload of the left ventricle and they described a relative wall thickness (RWT) of 0.41. The authors concluded that the relation of myocardial hypertrophy to exercise training is strengthened further by exercise blood pressure. However, unlike the pathologic hypertrophy resulting from hypertension, the triathlete’s heart would show “normal” diastolic LV-function.

The difference between triathletes and racing cyclists is that the thriathlete’s training does not only take place under strength/endurance conditions, but also under running conditions. Modified strength training also results in different changes in the cardiac structures of triathletes in comparison to professional racing cyclists ^51^. In a study including 14 professional cycle racers it was shown that the left ventricular muscle mass resulted in eccentric hypertrophy compared to concentric hypertrophy as also shown in our study. Therefore, the functional changes found in the cardiac structures for triathletes resemble the changes in runners ^52^. Sudden cardiac death of athletes is more common in male athletes ^27, 53^. The different causes of sudden cardiac death are silent coronary disease ^54^, hypertrophic cardiomyopathy ^55^ and arrhythmogenic right ventricular cardiomyopathy ^56^ ( Table 6). Maron *et al.* ^55^ described hypertrophic cardiomyopathy as common cause of sudden cardiac death (36%) in young athletes and 8% were presented with indeterminate LV-hypertrophy (possible HCM). The probability of the negative role of hypertrophy in athletes ^10^ and the problem of qualitative and quantitative relevance are under discussion ^57, 58^.

**Table 6.** Causes of sudden cardiac death in young athletes <35 years in %.

Causes of sudden cardiac death	Maron 2007 ^55^	Corrado 2003 ^56^	Solberg 2010 ^54^	Marijon 2011 ^27^
Aortic rupture	2.2	1.8	4.3	2
Aortic stenosis/cong. HD	1.8		4.3	6
ARVC	4	22		4
Channelpathies (QT, WPW)	3	1.8	8.7	12
Coronary artery anomalies	24	11	3.3	
Coronary disease	3	18	48	6
Dilatative CM	2	1.8		4
Hypertrophic CM	36	1,8	4.3	10
MVP	4	7.3		2
Myocarditis	5.4	9	22	4
Possible HCM	8			4
Riva muscle bridge	2.2	3.6		2
Unclear		1.8		36
	n = 1049	n = 55	n = 22	n = 50

cong. HD = Congenital Heart Disease. ARVC = Arrhythmogenic Right Ventricular Cardiomyopathy. QT = QT-Syndrome: Romano-Ward Syndrome, and Jervell-Lange-Nielsen-Syndrome. WPW: Wolff-Parkinson-White Syndrome. CM = Cardiomyopathy. HCM = hypertrophic Cardiomyopathy. MVP = Mitral Valve Prolapse.

### Left ventricular “fatigue”

Some papers have reported that excessive endurance training may cause negative remodelling of cardiac structures ^15, 59^. Predominantly marathons and Ironman-distance triathlons can cause a transient overload of the right ventricle ^59^. Fibrosis of the left ventricle in older runners was described as a possible cause of death ^24, 25^. Numerous investigations regarding the increase in bio-markers (mainly Troponin cTnI and NTproBnP) in runners of marathons ^60^ as well as triathlon ^61^ competitions have been conducted. A significant increase in bio-markers after the race was found in all those studies. Uniformly, this was considered as a proof of possible injuries to the heart muscle ^62^. Overall, the increase in bio-markers in athletes with intensive muscle work should not necessarily be interpreted as heart specific ^63^, because it also depends on the athlete’s weight ^64^ and may be associated with the myolysis (creatine kinase up to 10000 U/l after long-term running) ^65^. The discussion on this issue is ongoing ^17, 20, 66^.

### Limitations and future directions

The cross-sectional design of this study does not allow a causality regarding the negative role of EIAH in athletes to be established. Although our data suggest that left ventricular hypertrophy might be related to EIAH beyond the normal exercise-induced adaptation, confirmatory longitudinal work is necessary.

The results of this study support the authors’ subjective impression of daily practice and engagement in sports medicine over 15 years. We observe rhythm disorders in many cyclists and triathletes around the age of 50, and many of them have elevated blood pressure values during exercise. The probability of increasing stiffness of the aorta as an aging process supported by EIAH remains to be discussed. The present study attempts to analyse the probability of LVM and EIAH and should stimulate further follow-up investigations. It is a very important aim to prevent a potential fibrosis of the left atrium ^67^ or left ventricular myocardium in athletes ^16^ in order to avoid “negative cardiac remodelling” induced by exercise and to preserve the positive effects of physical activity ^12^. Approximately two million people participate in long-distance races in the United States annually ^68^ and there are only limited data regarding their exercise-induced blood pressure, which might be one of the main factors triggering cardiac events ^69, 70^.

## Conclusions

The relationship between myocardial hypertrophy and arterial blood pressure during exercise remains an open issue. The literature ^13, 42^ seems to suggest a clear relationship. The relevance of EIAH has to be examined in the future in consideration of serious reports ^8, 57, 58^. The cited authors suggested the isolated (without EIAH) exercise-induced hypertrophy as a substrate for sudden cardiac death or rhythm disorders. EIAH may enhance the “physiological” exercise-induced hypertrophy in a pathological way. Accordingly, the blood pressure values or EIAH should be thoroughly examined during routine or pre-event check-up.

The long training-times for Ironman-distances of triathletes with EIAH can lead to additional enlargement of the heart cavities ( Figure 2) and may trigger possible sudden cardiac death during triathlon competitions ^71^.

There is strong evidence that athletes have higher incidence of atrial fibrillation and bradyarrhythmias increasing with age ^21– 23^. We don’t know the definitive reasons for this, but EIAH and LVM might be one of the factors. Cases of early death in individual cases due by myocardial fibrosis are possible ^24, 25^. However, the general prevalence or incidence of EIAH in athletes is unknown. The problem of EIAH seems to be linked more to competitive athletes with vigorous training and mainly to males. It is known that low-intensity training ^72^ and aerobic exercise have a positive impact on blood pressure lowering ^73– 75^. The hypertensive or non-hypertensive response to exercise seems to be related to hereditary factors ^76^, to aging or to the individual arterial stiffness ^43^. It is crucial to define the people at risk and possibly start therapy ^77^. In our daily practice we treat the athletes at risk with low-dose ACE-inhibitors or AT _1_-blockers before training or competition. The dosage should be tested using an exercise test. Possible therapies for the prevention of fibrosis or atrial fibrillation have already been discussed ^23, 78^.

Further international, prospective, longitudinal studies on possible negative cardiac remodelling caused by EIAH and sport should be conducted. These studies could help to avoid the adverse effects of sport in people at risk. The overlap of EIAH and exercise-induced hypertrophy has the potential for increased QT-dispersion ^79^ and is a ticking clock for cardiac fatigue especially for middle aged men. Independent of all the competitive sporting activities with an enormous importance for hobby-athletes, media and industry, physical activity in general population is of fundamental importance ^11, 12^.

## Consent

All athletes provided written informed consent to voluntary testing of the performance and using the data for the study. Triathletes underwent their annual medical check-up or examination for planning their training, which would have been carried out in clinical routine in any case. A special approval by an ethics committee was not mandatory because of the study independent character of the examinations. The examinations were a part of clinical routine support of the triathletes. Pharmaceutical interventions in the triathletes were not affected by the study.

## Data availability

*figshare*: Data of exercise-induced arterial hypertension in triathletes, doi: http://dx.doi.org/10.6084/m9.figshare.1010160 ^80^
