# Supplementary material for: Exercise-induced arterial hypertension - an independent factor for hypertrophy and a ticking clock for cardiac fatigue or atrial fibrillation in athletes?
Source: F1000Res. 2014 May 12;3:105. [Version 1] doi: 10.12688/f1000research.4001.1 (PMC4118759; doi:10.12688/f1000research.4001.1)
Supplement: Peer review history

# Peer review: Referee response for version 1

**DOI:** 10.5256/f1000research.4288.r5134

**Published:** 2014-07-01

**Authors:** Marta Sitges, Maria Sanz de la Garza

**Affiliations:** 1 Thorax Institute, Universitat de Barcelona and Institut d'Investigacions Biomèdiques August Pi i Sunyer (IDIBAPS), Barcelona, Catalonia, Spain; 2 Hospital Clinic Barcelona, Barcelona, Catalonia, Spain

The study examined the potential relationship between exercise-induced arterial hypertension (EIAH) and left ventricular hypertrophy in 51 normotensive healthy male triathletes. For that purpose, they divided the study population into two groups according to left ventricular mass (LVM), choosing as cut-off point of significance left ventricular hypertrophy: 220g and analyzed the systolic blood pressure (BPs) in a cardiopulmonary exercise test on a cycle-ergometer. Triathletes with LVM> 220g showed significantly higher BPs at the anaerobic threshold and a trend to higher BPs values at the aerobic threshold and at maximum exercise level.

They concluded that significant left ventricular hypertrophy is associated with higher blood pressure at the anaerobic and aerobic threshold and suggest that endurance athletes with EIAH could benefit from preventive antihypertensive treatment.

**Comments:**

1. The assessment of blood pressure response to exercise has been typically limited to systolic values. Classic studies such as the Framingham study and preliminary studies from our group ^1^ ^2^ have identified the diastolic pressure response to exercise as the best predictor of late development of hypertension. It would have been interesting to perform a complete analysis of the blood pressure response during exercise, including both systolic and diastolic values.
2. The authors relate EIAH to the development of atrial fibrillation and in fact, this association is used in the title. The study analysis though, did not demonstrate this association, as clinical or electric data related to atrial fibrillation are not analyzed. Athletes with LV mass >220g showed a significantly higher left atrial diameter and a tendency to a higher left atrial volume but all values ​​are within normal limits. Moreover, the author connects EIAH with aorta stiffness but no differences between groups were documented in LVOT and all values are also within normal limits.
3. The authors claim that they did not find any particular right ventricular remodeling, but the only reported data concerning RV analysis are insufficient: fractional RV area and RV diameter measurement in the parasternal view. On the other hand, this later measurement was significantly larger in the group with LV> 220g.
4. We would like to point out two potential typographic errors in Table 1: the data on weight and height seem to be exchanged and the data concerning the systolic diameter of the left ventricle seems indeed to be related to diastolic diameter.
5. How was the cut-off point of LV mass 220g selected? Why didn’t the authors choose an indexed value of LV mass by body size? At least, this should be acknowledged as a limitation of the study.
6.  It is well known that measuring blood pressure at high loads of physical exercise might be challenging. The methodology of the determination of blood pressure during exercise should be better described.

 

Overall a state of the art and an interesting line of investigation, with illustrative results but it may be still preliminary to claim an association with atrial fibrillation and advise preventive antihypertensive treatment in athletes.

We have read this submission. We believe that we have an appropriate level of expertise to confirm that it is of an acceptable scientific standard.

## References

[1] YzaguirreIDomenechMGrazioliGPiRGutierrezJAVinusaABrugadaJCocaAStigesM: Exaggerated blood pressure response in young individuals during the stress test: which is the best methodology to predict the incidence of hypertension or cardiovascular disease?. *Eur. Heart J*.2013;34: 10510.1093/eurheartj/eht309.P3402

[2] GrazioliGYzaguirreIDomenechMComellasCCifuentesMJCocoaAGutierrezJBrugadaJStigesM: Utility of blood pressure measured during an exercise as a risk factor for new onset hypertension. *Eur. Heart J*.2013;34: 10510.1093/eurheartj/eht309.P3381

# Peer review: Referee response for version 1

**DOI:** 10.5256/f1000research.4288.r4745

**Published:** 2014-06-17

**Authors:** Céline Fiset

**Affiliations:** 1 Montreal Heart Institute and Faculty of Pharmacy, Université de Montréal, Montréal, QC , Canada

This study examined the impact of exercise-induced arterial hypertension (EIAH) on cardiac hypertrophy in normotensive healthy Ironman athletes. The hypothesis they wish to test is that elevated blood pressure values during exercise promote left ventricular hypertrophy in athletes and is one important factor causing “exercise-induced” cardiac fatigue. This is an interesting and current research topic considering that more and more evidence suggest that intense exercise training can lead to harmful cardiac remodelling in athletes who are otherwise healthy.

In support of a negative role for EIAH, findings reported in this cross-sectional study indicate that left ventricular hypertrophy is associated with higher arterial blood pressure in the athletes examined. This paper provided interesting information; however, as acknowledged by the authors further research is required to clearly establish the causal relationship between EIAH and left ventricular hypertrophy. Moreover, based on the results presented in this study, it seems precipitate to suggest that endurance athletes with EIAH may require a therapeutic intervention such as low-dose ACE-inhibitors or AT1-blockers before training or competition to prevent exercise-induced cardiac hypertrophy.

The authors report that the problem of EIAH is mostly encountered in men. The authors may want to consider that this apparent sex-specific regulation of EIAH is presumably a “gender”-related differences rather than a “sex”-related difference; considering that it is likely that less women will be inclined to enrol in vigorous training such as Ironman-distance triathlon, suggesting that biological and/or physiological differences are probably not involved in the male prevalence of EIAH.

The title of the paper is somewhat misleading as the data reported in this study is not directly related to atrial fibrillation. Similarly, the study does not address the relationship between EIAH and bradycardia or QT dispersion. Furthermore, given that no data directly addressed the link between EIAH and AF, bradycardia and QT dispersion, the authors should consider revising their text to not over extrapolate their findings.

I have read this submission. I believe that I have an appropriate level of expertise to confirm that it is of an acceptable scientific standard.
